# Reasons for discontinuing urate-lowering treatment in community-dwelling adults with gout: results of a primary care-based cross-sectional study

**DOI:** 10.1093/rap/rkab022

**Published:** 2021-04-11

**Authors:** Dalia Elmelegy, Abhishek Abhishek

**Affiliations:** 1 Academic Rheumatology, University of Nottingham, Nottingham, UK; 2 Physical Medicine, Rheumatology and Rehabilitation Department, Faculty of Medicine, Tanta University, Egypt; 3 Nottingham NIHR-BRC, Nottingham, UK

**Keywords:** gout, allopurinol, urate-lowering therapy, persistence

## Abstract

**Objectives:**

The aim was to examine the prevalence of urate-lowering treatment (ULT) in community-dwelling adults with gout and the reasons for drug discontinuation.

**Methods:**

Adults with gout living in the East Midlands, UK, were mailed a postal questionnaire by their general practice surgery. It enquired about demographic details, co-morbidities, number of gout flares in the previous 12 months, current ULT and the reasons for discontinuing ULT if applicable. The number (percentage), median [interquartile range (IQR)] and mean (s.d.) were used for descriptive purposes. The Mann–Whitney *U* test and χ^2^ test were used for univariate analyses. STATA v.16 was used for data analysis. Statistical significance was set at *P* < 0.05.

**Results:**

Data for 634 gout patients [89.3% men, mean (s.d.) age 64.77 (12.74) years)] were included. Of the respondents, 59.8% self-reported taking ULT currently, with the vast majority (95.6%) taking allopurinol. Participants self-reporting current ULT experienced fewer gout flares in the previous 12 months than those who did not self-report current ULT [median (IQR) 0 (0–2) and 1 (0–3), respectively, *P* < 0.05]. One hundred and seven participants (16.9%) self-reported ULT discontinuation previously. The most commonly cited reasons for this were side-effects (29.7%), being fed up with taking tablets (19.8%) and lack of benefit from treatment or ULT-induced gout flares (19.8%). Treatment being stopped by the general practitioner without a clear reason known to the participant (15.8%) was another common report.

**Conclusion:**

This study identified patient-, physician- and treatment-related barriers to long-term ULT. These should be addressed when initiating ULT and during regular review. Further research is required to confirm these findings in other populations.

Key messages

- Side-effects are the most common reason for non-persistence with urate-lowering treatment.
- Being fed up with treatment and lack of improvement were other common patient factors for discontinuation of urate-lowering treatment.
- Individualized patient education, screening for side-effects and periodic follow-up should be considered upon initiating urate-lowering treatment.

## Introduction

Gout affects 2.5% adults in the UK, with higher prevalence reported from elsewhere in the world [1]. Its manifestations range from intermittent flares to tophi and joint damage [2]. Gout flares can be managed with anti-inflammatory drugs, such as NSAIDs, colchicine or CSs [2]. However, the goal of gout treatment is to dissolve all monosodium urate crystal deposits using long-term treat-to-target urate-lowering treatment (ULT), thereby preventing flares and resolving tophi if present [2]. Many patients adhere poorly to ULT and discontinue therapy before experiencing symptomatic improvement on patient-centred outcomes, such as gout flares [3]. Side-effects or apprehension about developing them, cost of treatment, unwillingness to take life-long treatment, polypharmacy and gout flares triggered by ULT emerged as some of the reasons for drug discontinuation in the interview studies that explored patients’ perception of gout and their experience of receiving treatment for it [4–9]. However, these studies explored a wide range of issues and did not focus specifically on the reasons why ULT is discontinued. Additionally, although qualitative studies give an indication of the barriers to long-term ULT, they do not inform about the contribution of each factor to the overall problem.

It is important to identify the common reasons for which ULT is discontinued in order that they can be addressed proactively in routine clinical care of gout patients. Thus, the objectives of this study were to examine the prevalence of ULT in community-dwelling adults with gout and to identify the reasons for non-persistence with ULT. We explored whether the reasons for discontinuation of ULT differed according to age, sex and co-morbidities.

## Methods

This was a primary care-based cross-sectional study. Participants were adults (age >18 years) diagnosed with gout or ever prescribed a ULT according to the general practice (GP) electronic medical and prescription records, registered with one of the 22 GP surgeries in the East Midlands region of the UK that are taking part in this study.

Potential participants were mailed a questionnaire by their GP surgery, enclosing a pre-paid envelope addressed to the research team at Academic Rheumatology, City Hospital Nottingham, with instructions to return the completed questionnaires to the research team directly. The questionnaire enquired about their demographic details, physician-diagnosed co-morbidities, age of gout onset, number of gout flares in the previous 12 months, current and previous ULT, and reasons for discontinuing ULT if applicable. The participants were asked to select as many reasons for discontinuing ULT from among five common reasons for discontinuing treatment based on literature review and patient and public involvement input (Supplementary Data S1, available at *Rheumatology Advances in Practice* online). Additionally, other reasons for drug discontinuation could be provided as free text. The free-text data were grouped together into themes. Self-report of physician-diagnosed hypertension, hypercholesterolaemia, coronary heart disease, diabetes, stroke, renal stone, chronic kidney disease and OA was added to calculate a co-morbidity score that ranged from zero to eight.

Socio-economic deprivation was assessed using the index for multiple deprivation (IMD) based on the participant’s postcode. The UK is divided into 1.8 million postcodes, and each postcode typically has a small number of dwellings. The IMD takes account of seven different domains of deprivation and provides a composite score, with higher scores indicating worse deprivation. Each area in England is ranked from least to most deprived. The rank score is converted into deciles, with higher values indicating more deprivation. The IMD scores for 2019 were obtained from imd-by-postcode.opencomunities.org/imd/2019.

Continuous data were assessed for their distribution. The mean (s.d.), median [interquartile range (IQR)] and number (percentage) were used for descriptive purposes for normally distributed, non-normally distributed and categorical data, respectively. Parametric (*t* test, χ^2^ test) or non-parametric (Mann–Whitney *U* test) hypothesis tests were used for univariate analysis depending on data distribution. Logistic regression was used to examine the association between ULT status (currently on treatment *vs* treatment stopped) and the number of co-morbidities (ranging from zero to eight). A value of *P *<* *0.05 was defined as statistically significant. Data were analysed using STATA v.16. This study was approved by the Nottingham NHS Research Ethics Committee (18/EM/0324), and all participants gave written informed consent.

## Results

Two thousand four hundred and eleven people with gout registered with 22 GP surgeries in the East Midlands, UK were mailed the postal questionnaires by their GP, and 634 replies (26.3%) were received. Their mean (s.d.) age and disease duration were 64.77 (12.74) and 13.83 (13.27) years, respectively; 89.3% respondents were men. The median (IQR) number of self-reported gout flares in the previous 12 months was 0 (0–2). The prevalence of self-reported physician-diagnosed co-morbidities was as follows: hypertension, 44.3%; hypercholesterolaemia, 25.1%; diabetes, 11.2%; coronary heart disease, 9.2%; and chronic kidney disease, 6.5%. The median (IQR) co-morbidity and IMD scores were 2 (0–2) and 7(4–9), respectively. Of the participants, 59.8% self-reported taking ULT currently, with the vast majority on allopurinol treatment (95.6%). No participant self-reported taking combination ULT. The median (IQR) number of gout flares in those currently taking and not taking ULT were 0 (0–2) and 1 (0–3), respectively (*z* = 3.49, *P* = 0.0005, Mann–Whitney *U* test).

**Fig. 1:**
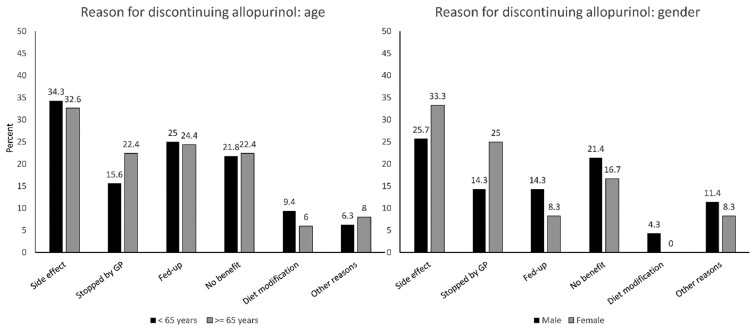
Reason for discontinuation of urate-lowering treatment according to age and sex GP: general practitioner.

Of the respondents, 101 (15.9%) self-reported discontinuing allopurinol previously (Supplementary Fig. S1, available at *Rheumatology Advances in Practice* online) and 81 participants provided reason(s) for its discontinuation. The commonest reasons for stopping allopurinol treatment were side-effects, no benefit from ULT, and being fed up with taking tablets (Table 1). Only five participants self-reported discontinuing allopurinol owing to ULT-triggered gout flares, and they were included in the no benefit from ULT category. The reasons were similarly distributed in those <65 and ≥65 years of age, and in men and women (Fig. 1). Two participants self-reported stopping benzbromarone owing to its side-effects, and four participants self-reported discontinuing febuxostat. Of these, three provided reasons for drug discontinuation [side-effects (*n* = 1), being fed up with taking tablets (*n* = 1) and no improvement in gout (*n* = 1)].

**Table 1:** Reasons for discontinuing allopurinol

Reason^a^	Participants (*n* = 81)^b^
Side-effects	30
Stopped by GP with no clear reason	16
Fed up with taking tablets	20
No benefit or ULT triggered flares	20
Dietary modification or supplements	7
Flare prophylaxis or flare treatment alone	5
Improvement in gout	4
Participating in clinical trial	1

a Participants could report more than one reason. ^b^One hundred and three reasons were self-reported by 81 participants. GP: general practitioner; ULT: urate-lowering treatment.

The cumulative co-morbidity score was not associated with discontinuing ULT (odds ratio 0.81, 95% CI 0.63, 1.05). Side-effects of ULT (33.33% *vs* 39.29%) and being fed up with taking tablets (29.63% *vs* 25%) were numerically equally common in the participants with zero or one and with two or more co-morbidities, respectively, who self-reported discontinuing ULT and provided a reason for it in their questionnaire response. However, stopping treatment owing to lack of improvement in gout and being stopped by the GP without a clear reason given to the participant was reported by 31.48% *vs* 7.14% and 5.55% *vs* 28.57% participants with zero or one and with two or more co-morbidities, respectively, who self-reported discontinuing ULT and provided a reason for it (*P* < 0.001).

## Discussion

The main finding of this study is that ULT discontinuation is multifactorial and is influenced by drug, disease, patient and health-care provider factors. The common reasons for discontinuing ULT identified in our study were side-effects, being fed up with taking long-term treatment and lack of improvement in gout. Treatment being stopped by the GP without an explanation was another important factor. Additionally, a very few participants discontinued ULT owing to improvement in gout; choosing to use lifestyle interventions, flare prophylaxis or flare treatment. Co-morbidities, but not age and sex, influenced the reason(s) for discontinuing ULT. People with no or one co-morbidity were more likely to discontinue treatment for lack of benefit, whereas those with two or more co-morbidities were more likely to have their treatment discontinued by the GP without a clear reason known to the participant. Finally, we observed that the point prevalence of ULT in these 22 GP practices was slightly <60%.

Approximately 30% of participants who discontinued ULT did so because of side-effects. This is consistent with the findings of previous qualitative studies from the USA and the UK, in which side-effects emerged as important concerns for people with gout and as a reason for discontinuing treatment or reducing the dose [4–9]. Some participants in the previous studies were also concerned that long-term treatment might result in them accumulating even more side-effects over time [5, 9].

Lack of improvement or ULT-triggered gout flares were the other common reasons for drug discontinuation. This is consistent with the results of previous interview studies, in which these factors emerged as common reasons for discontinuation of treatment [4–6]. Participants with no or one co-morbidity were more likely to discontinue treatment for this reason than those with two or more co-morbidities. This might be attributable to an expectation mismatch, because patients without underlying health issues might expect a rapid improvement in their condition. Being fed up with taking tablets long term was also a common reason for drug discontinuation. This is consistent with the findings of a previous study, in which many patients expressed unwillingness to take long-term medications for gout because they did not feel that long-term treatment was required for it [5, 8, 9].

GPs discontinuing the treatment without a clear reason explained to the participant emerged as a less common reason. However, it was significantly more common in people with two or more co-morbidities than in those with no or one co-morbidity. This raises the possibility that ULT is being stopped owing to concerns about drug interactions and/or side-effects and to reduce polypharmacy. However, this could occur owing to patients forgetting to ask for their repeat prescriptions and the GP then discontinuing the prescription of drugs that were not collected on multiple occasions. Indeed, forgetfulness was identified as a reason for poor adherence to ULT in two previous studies [4, 6]. Stopping treatment because participants felt their gout was cured owing to not having experienced a flare for a prolonged period emerged as a reason for drug discontinuation in one interview study, but was relatively infrequent in the present study [5]. In previous interview studies from the USA, cost of treatment emerged as a reason for drug discontinuation. It is not surprising that this did not appear as a reason for drug discontinuation in the UK, where health care is free at the point of delivery [4, 6]. Polypharmacy and difficulty in swallowing tablets emerged as reasons for poor adherence in previous qualitative studies [4–7]. However, these were not self-reported as reasons for discontinuing ULT in our study. Preference for alternative therapies was identified as a reason for poor adherence to ULT previously; however, our study suggests that this is relatively uncommon.

Slightly <60% of respondents self-reported current ULT. This is higher than the prevalence of ULT in the UK reported previously [10, 11]. This might be a regional phenomenon or attributable to an increase in ULT prescriptions in the UK. Further nationwide studies are needed to confirm these findings. However, in keeping with national trends, the vast majority of patients self-reporting current ULT were taking allopurinol [10, 11].

Our study has implications for clinical practice. Patients with gout commenced on ULT should be reviewed periodically for side-effects, and their concerns should be addressed. Patients initiating ULT should receive individualized education about gout and its treatment. This should specifically address: (a) the need to persevere with treat-to-target ULT in the long term (e.g. for ≥2 years) before benefits such as reduction in flare frequency and improvement in quality of life are felt [12]; (b) the risk of gout flares triggered on initiation of ULT and measures such as flare-prophylaxis that might be used to minimize these risks [13]; and (c) the fact that serum urate will increase to pre-ULT levels within weeks of discontinuing treatment and might result in recurrent gout flares if treat-to-target ULT is not re-introduced [14].

This is the first study to examine quantitatively the reasons for non-persistence with ULT. Strengths include primary care-based recruitment, structured self-report questions and an option to provide free-text data. However, this study has several limitations. The reasons for discontinuation of allopurinol were self-reported by only 81 participants, and the study was conducted in only one region of the UK, which limits the generalizability of the study. The reasons for discontinuing ULT and the number of gout flares in the previous 12 months were self-reported and might be affected by biased recall. In addition, self-report of ULT might also have inflated estimates of persistence, and we did not have access to the prescription records to validate the self-reported data. Also, the demographic data for non-respondents was not available to the research team owing to confidentiality and data protection. This prevents any comparisons from being made between respondents and the original target population. Moreover, we did not collect data on adherence to ULT and the use of flare prophylaxis. The questionnaire respondents comprised a more deprived sample than the UK average population. However, the East Midlands is more deprived than the UK average, and the deprivation scores of respondents were comparable to those from the region. Finally, this study was conducted in the East Midlands, UK, and results might differ in other populations.

In summary, this study identified several reasons for non-persistence with ULT and estimated their prevalence. Previous research has demonstrated that addressing these factors proactively results in improved persistence with ULT and better patient-centred outcomes (e.g. fewer gout flares) in a research setting [9, 12, 15, 16]. Further research is required to find out whether adopting these strategies in routine clinical care improves persistence with ULT and patient-centered outcomes.

*Funding*: This work was supported by the Versus Arthritis research grant (grant number 21506 to A.A.) and the Newton-Mosharafa Fund (to D.M. and A.A.).

*Disclosure statement*: A.A. has received departmental research grants from AstraZeneca and Oxford Immunotech, speaker bureau fees from Menarini, scientific meeting attendance support from Pfizer, Royalties from UpToDate and Springer, and has consulted for Iflazome unrelated to this work. The other author has declared no conflicts of interest.

## Data availability statement

The data that support the findings of this study are available from the corresponding author, D.M., upon reasonable request.

## Supplementary data

Supplementary data are available at *Rheumatology Advances in Practice* online.

## Supplementary Material

rkab022_Supplementary_Data
