# Supplementary material for: Reasons for discontinuing urate-lowering treatment in community-dwelling adults with gout: results of a primary care-based cross-sectional study
Source: Rheumatol Adv Pract. 2021 Apr 11;5(2):rkab022. doi: 10.1093/rap/rkab022 (PMC8195916; doi:10.1093/rap/rkab022)

**Supplementary Data S1. Questionnaire**

Place an **X** to tell us if you take any of the following medicines currently?

Allopurinol

Febuxostat

Benzbromarone

None of the above

Now, please place an **X** to tell us tell us if you have ever been started on any of the following medicines, only for it to be stopped later on.

Allopurinol

Febuxostat

Benzbromarone

None of the above

Please place an **X** to select the main reasons why the medicine was stopped. You may select more than one option

|  | *It caused gout attacks* | *It did not improve my gout* | *I had*  *side effects* | *My GP stopped prescribing for no reason* | *I got fed-up of taking tablets* |
| --- | --- | --- | --- | --- | --- |
| Allopurinol |  |  |  |  |  |
| Febuxostat |  |  |  |  |  |
| Benzbromarone |  |  |  |  |  |

Other reasons (please write here):_____________________________________________

**Supplementary Figure S1.** **Flowchart of the participants.** ULT: Urate Lowering Treatment.


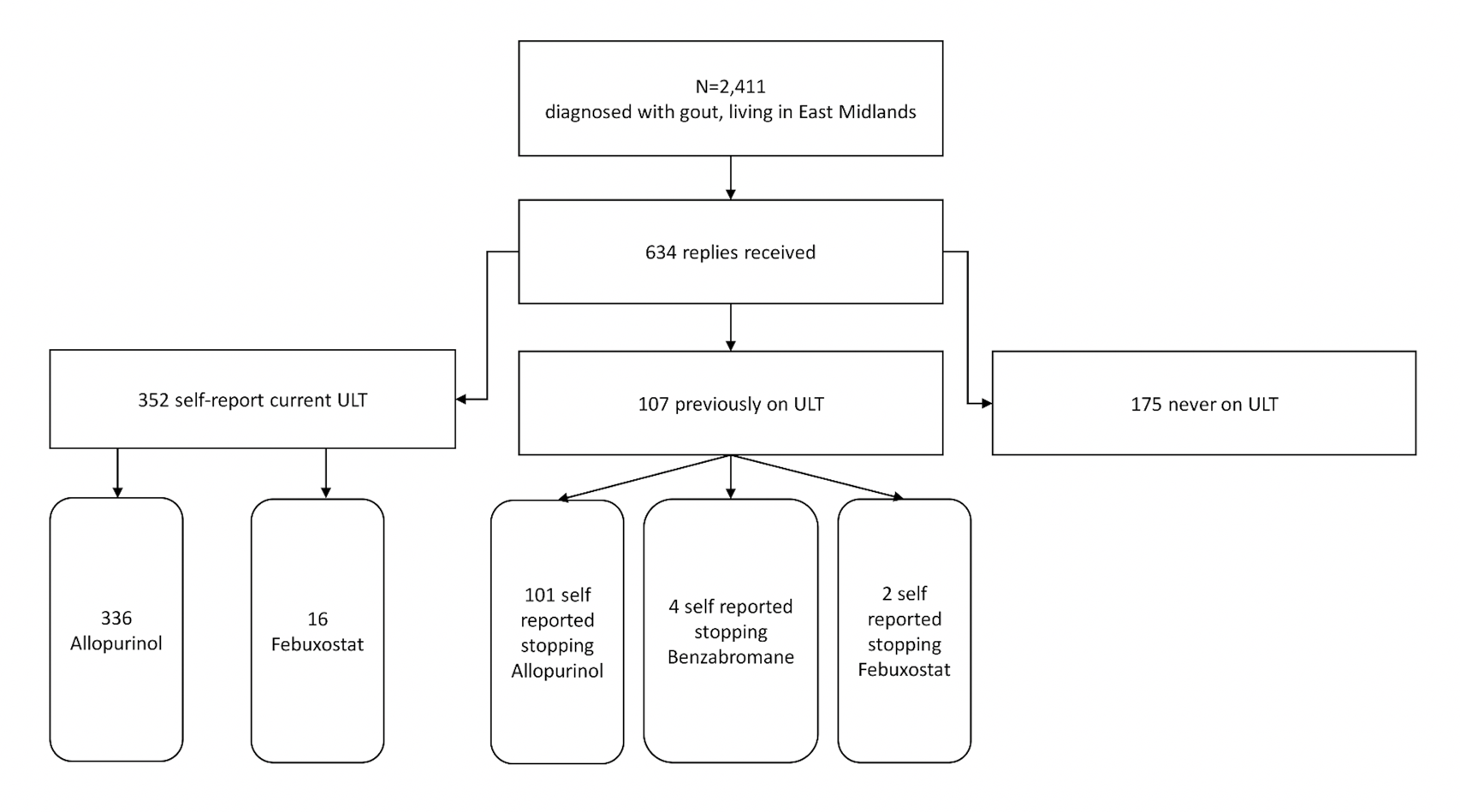

Supplement: rkab022_Supplementary_Data [file rkab022_supplementary_data.docx]
